# A novel quantitative analysis method for idiopathic epiretinal membrane

**DOI:** 10.1371/journal.pone.0247192

**Published:** 2021-03-17

**Authors:** Davide Allegrini, Giovanni Montesano, Stefania Marconi, Nicoletta Rosso, Giovanni Ometto, Raffaele Raimondi, Ferdinando Auricchio, Panagiotis Tsoutsanis, Francesco Semeraro, Matteo Cacciatori, David P. Crabb, Mario R. Romano

**Affiliations:** 1 Department of Biomedical Sciences, Humanitas University, Milan, Italy; 2 Department of Ophthalmology, Humanitas Gavazzeni–Castelli, Bergamo, Italy; 3 City, University of London—Optometry and Visual Sciences, London, United Kingdom; 4 NIHR Biomedical Research Centre, Moorfields Eye Hospital NHS Foundation Trust and UCL, Institute of Ophthalmology, London, United Kingdom; 5 Department of Civil Engineering and Architecture, University of Pavia, Pavia, Italy; 6 Ophthalmology Service, Istituti Ospitalieri di Cremona, Cremona, Italy; 7 Department of Medical and Surgical Specialties, Radiological Sciences and Public Health, Eye Clinic, University of Brescia, Brescia, Italy; Massachusetts Eye & Ear Infirmary, Harvard Medical School, UNITED STATES

## Abstract

**Purpose:**

To introduce a novel method to quantitively analyse in three dimensions traction forces in a vast area of the ocular posterior pole.

**Methods:**

Retrospective analysis of 14 eyes who underwent peeling surgery for idiopathic, symptomatic and progressive epiretinal membrane. The technique measures the shift in position of vascular crossings after surgery from a fixed point, which is the retinal pigmented epithelium. This shift is defined as the relaxation index (RI) and represents a measure of the postoperative movement of the retina due to released traction after surgery.

**Results:**

Best-corrected visual acuity was significantly better than baseline at all follow ups while the RI had its maximum value at baseline. Moreover, we found a significant correlation between best-corrected visual acuity at 6 months and RI at baseline.

**Conclusion:**

While all previous published methods focused on bi-dimensional changes observed in a small region, this study introduces a three-dimensional assessment of tractional forces. Future integration of RI into built-in processing software will allow systematic three-dimensional measurement of intraretinal traction.

## Introduction

Epiretinal membrane (ERM) is a fibrocellular proliferation at the vitreoretinal interface above the inner limiting membrane (ILM) [1]. The prevalence of ERM increases with age and is reported between 2,2% in a population from Beijing (China) without a significative difference in patients that underwent cataract surgery and 28,9% in a multiethnic United States population with higher rates in patients that underwent cataract surgery and in patients of Chinese ethnicity [2–4]. A key element in ERM development is the fibrotic process which is sustained by collagen deposition and transdifferentiation into myofibroblast of retinal Müller cells, retinal pigmented epithelium (RPE) cells and hyalocytes [5–7]. The result is a semitranslucent, thick and contractile membrane that exerts a tractional force tangential to the retinal plane leading to macular puckering [4]. Patients symptoms range from being completely asymptomatic to complaining of metamorphopsia and loss of vision depending on macular involvement [4]. Disease progression leads to gradual but mild vision loss, a retrospective study reported that 21% of patients required surgery at 4 years if baseline visual acuity was ≥ 20/40 [8]. Treatment options consist of mainly in watchful waiting or vitrectomy surgery with peeling of the membrane [9]. The time point in which irreversible damage occurs is currently not clear, this causes uncertainty about surgical timing and an unevenness of protocols between centers. A classification by Gass [10] based on fundus appearance has been widely used to stage the disease. However, the introduction of spectral domain optical coherence tomography (SD-OCT) technology dramatically improved diagnosis and led to multiple OCT-based classifications. Hwang et al. [11] introduced a system grounded on foveal characteristics and validated with multifocal electroretinography (mERG) to demonstrate the functional differences between stages. Konidaris et al. [12] proposed a classification based on vitreous state and morphology, but validation was never carried out, thus this classification remains of uncertain clinical usefulness. Stevenson et al. [13] suggested the inclusion of central foveal thickness and inner segment ellipsoid band integrity as key morphologic parameters. Lastly, Govetto et al. [14] introduced a classification system with four stages based on the presence of the foveal pit and the integrity of outer and inner retinal layers. These classifications can be useful to link morphological features of the retina to postoperative prognosis. However, they mostly rely on qualitative descriptions of visible alterations. A more informative characterization of the disease could come from a quantitative and three-dimensional analysis of the retinal traction caused by the ERM.

In this study we introduce the Relaxation Index (RI) which allows a three-dimensional quantitative analysis of retinal tissue release, at different time points after removal of ERM in eyes affected by progressive epiretinal traction. We also relate the amount of relaxation to the functional visual outcome of the patients.

## Methods

### Sample description

The electronic medical records of the Ophthalmology Department of Humanitas Gavazzeni-Castelli Hospital of Bergamo and at the Ophthalmology Service ASST of Cremona were queried to identify all patients 18 years or older affected by idiopathic, symptomatic and progressive epiretinal membrane (ERM) that underwent peeling surgery between January and June 2019 (n = 142). The progression was defined as decrease of visual function, the presence of concomitant metamorphopsia and increased central macular thickness secondary to epiretinal traction. Exclusion criteria were: myopia greater than 6 diopters (n = 21), a history of mayor ocular surgeries not including cataract surgery (n = 15), macular edema secondary to vascular and tractional diseases (n = 6), uveitis (n = 3), diabetic retinopathy (n = 28), age-related macular degeneration (n = 16), complete follow up data not available (n = 53).

Therefore, we retrospectively analyzed 14 eyes from 14 patients who underwent peeling of the ERM and the ILM to treat visually significant ERM. Informed consent before surgery was obtained from all subjects, this study conformed to the Declaration of Helsinki and ethics approval was approved by the Ethics Committee of Humanitas Gavazzeni Hospital with the protocol number 42/20 GAV.

Surgeries were performed by two different surgeons (M.R.R.) and (M.C.). All patients were examined at one, three and six months after surgery. At all visits, best corrected visual acuity (BCVA) was measured in decimals. In four cases, the peeling was coupled with cataract surgery (Phaco-Peeling), the remaining eight patients were already pseudophakic.

### Relaxation index

The RI measures in, millimetres (mm), the shift in position of a vascular crossing after surgery from a fixed reference point, defined as the vertical projection of that same crossing onto the RPE at the last visit. This shift represents a measure of the postoperative movement of the retina due to released traction after surgery. For this analysis, we assumed that the last visit at 6 months was the maximally relaxed state. The RI used for the analysis is the average of the shifts of all the crossings identified by the graders for a given scan (see later).

**Fig 1:**
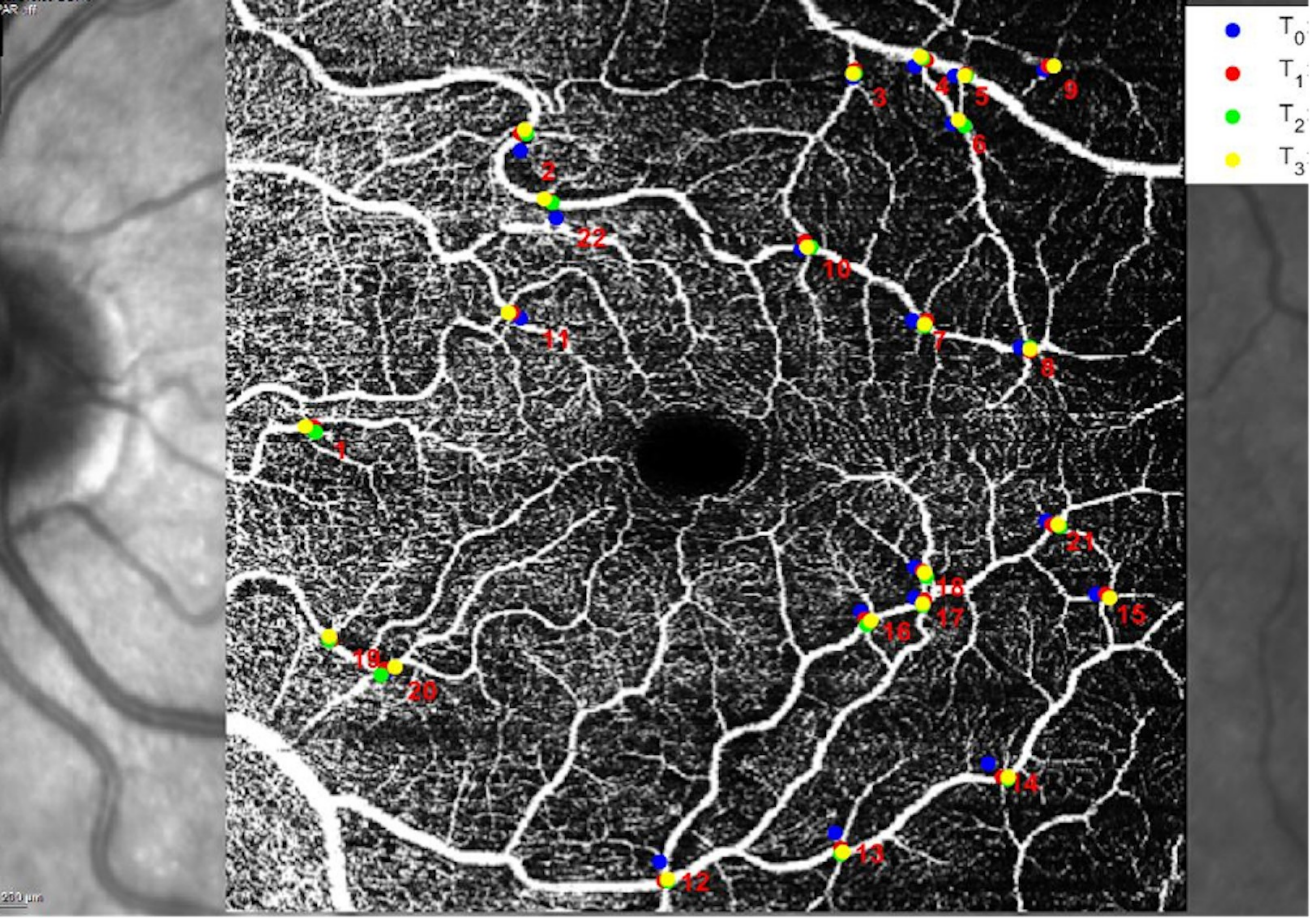
Example of vessel crossings marks at different timepoints on an elaborated OCTA scan. T_0_ = baseline (pre-op), T_1_ = 1 month post-op, T_2_ = 3 months post-op, T_3_ = 6 months post-op.

The RI Index of the retinal tissue was measured using the OCT angiography (OCT-A) scans. A Spectralis SD-OCT, (Heidelberg Engineering, Germany) was used to acquire OCT-A scans of the posterior pole at all time points with a field of view of 30°, using the follow-up mode. This employs a fundus tracking technology to ensure that OCT acquisitions are performed at the same location on the retina. The reference image is an infrared (IR) fundus picture paired with the OCT scans. Axial length was measured with a LS 900 TC optical low-coherence reflectometry biometer (Haag-Streit Diagnostics, Koeniz, Switzerland). A custom software was developed in Matlab (The MathWorks, Natick, USA), to enable a manual marking of any number of vessels crossing on OCT-A images acquired at different times. The software provides the coordinates of each selected crossing and computes their distances (Fig 1). Two independent graders (N.R. and M.C.) manually marked clearly identifiable vessel crossings on the baseline OCT-A. The graders then identified the same crossings on the aligned follow-up scans, so that their positions could be tracked in time. For all crossings, corresponding retinal thickness values were manually recorded using the Heidelberg Eye Explorer software, which computes thickness as the difference between ILM and RPE segmentations. OCT-A scans at all time points were studied for each of the 14 patients. All planar distances were corrected for ocular magnification using the axial length values and the schematic eye developed by Drasdo and Fowler [15].

**Fig 2:**
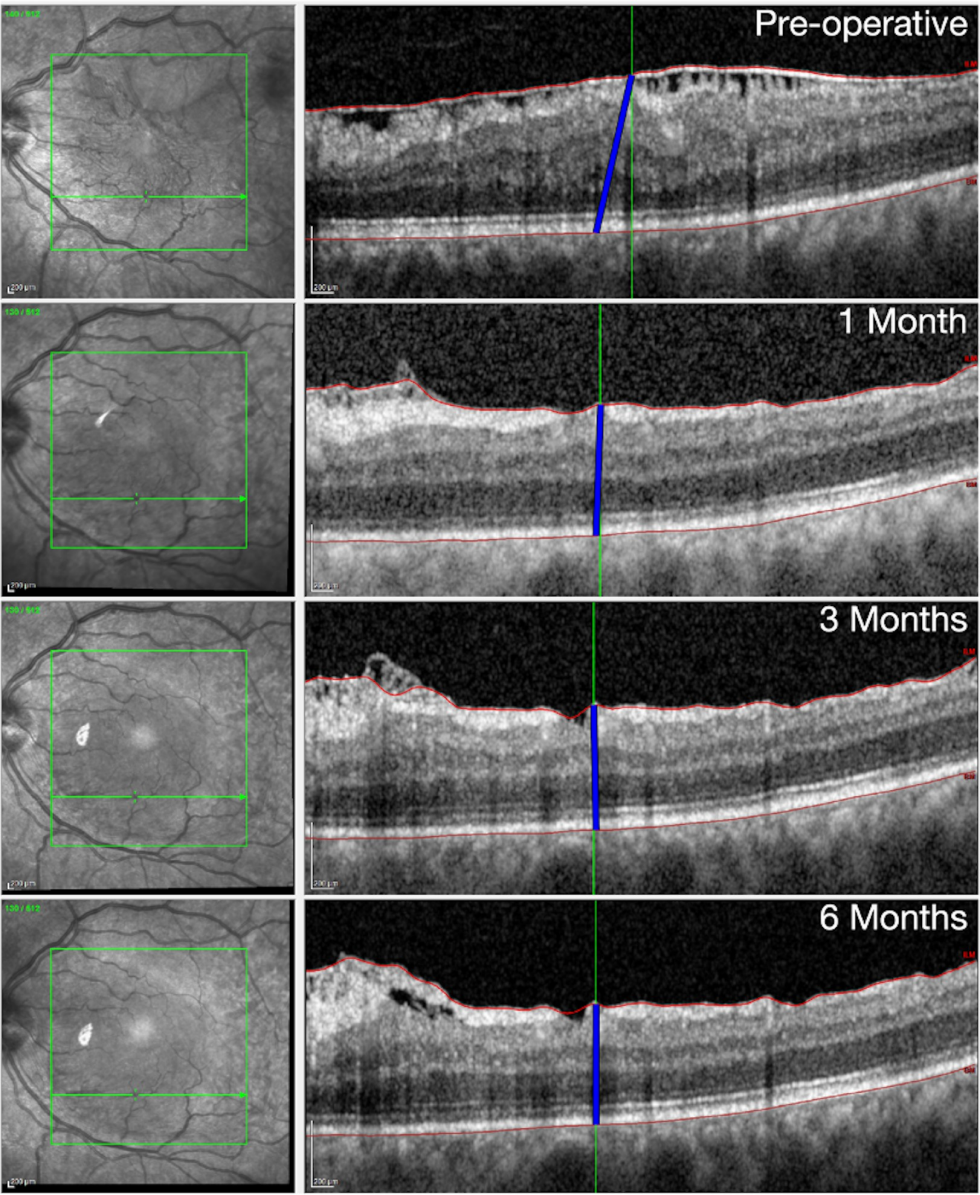
Example of how the position of a tracked vessel crossing changes its positions after surgery. The blue line connects the position on the Retinal Pigment Epithelium corresponding to the final position of the vessel crossing to its current position at the level of the inner limiting membrane over time. The length of the segment represented by the blue line is the Relaxation Index. Notice that this is a schematic representation on one B-scan but the actual calculation accounted for the length of the segment in the three dimensional volume scan.

We speculate that we can consider the scan at 6 months post-op (last available follow-up) as the maximum retinal relaxed state that was observed after surgery. Assuming that the outer retina is anchored to the RPE [16] so that no horizontal sliding of the retina is possible, the stretch of the tissue over time can be measured as the distance between the position at 6 months of RPE at a specific vessel crossing and the different positions of the same crossing over time (Fig 2). The length of this connecting segment represents the RI of the retina due to the superficial traction from the ERM, which is maximum in the pre-operative scan (baseline). The RI uses position changes of retinal vessels to describe the movement of the innermost retinal surface relative to the RPE. In the maximum relaxed state (6 months), it is simply the retinal thickness corresponding to each crossing. At each time point, the mean RI is calculated as the average of all measurements in each scan. In practical terms, the planar distance from the final position and the local thickness at each point in time represented the two orthogonal sides of a right triangle. Hence, the length of the segment was calculated as the squared root of the sum of squares of the planar distance from the final position at 6 months and the local thickness at each time-point for any individual vessel crossing.

### Statistical analysis

The inter-grader agreement was measured using a Bland-Altman plot and the 95% limits of agreement. Since each grader could select a different number of vessel crossings, the agreement was measured on the mean RI. For subsequent analyses, we used the average of the two mean RI from each grader.

The changes in BCVA and RI over time were explored using linear mixed effect models using the lme4 package for R [17]. Random effects were used to account for repeated measurements over time on the same eye. The fixed effect predictor was time, used as a categorical factor. These models were used to compare values at different follow-up visits with the baseline visit. Other comparisons that were not based on repeated measurements were performed with simple linear regressions. In particular, we studied the correlation between baseline RI and baseline BCVA using a univariate linear regression and the correlation between baseline RI and BCVA at 6 months using a multivariate linear regression that included the baseline BCVA as a covariate. The significance threshold was set at 0.05. The p-values were obtained from a t-type statistic for either the pair-wise differences between the time-points or for the regression coefficients. For pairwise comparisons between different time-points, the p-values were corrected for multiple (N = 4) tests using the Bonferroni-Holm method from the package lsmeans for R [18].

## Results

The average age at baseline was 70 ± 5 yeas (Mean ± Standard Deviation). The preoperative BCVA was 0.4 ± 0.1 (range 0.2–0.6). and the axial length was 23.9 ± 1 mm.

**Fig 3:**
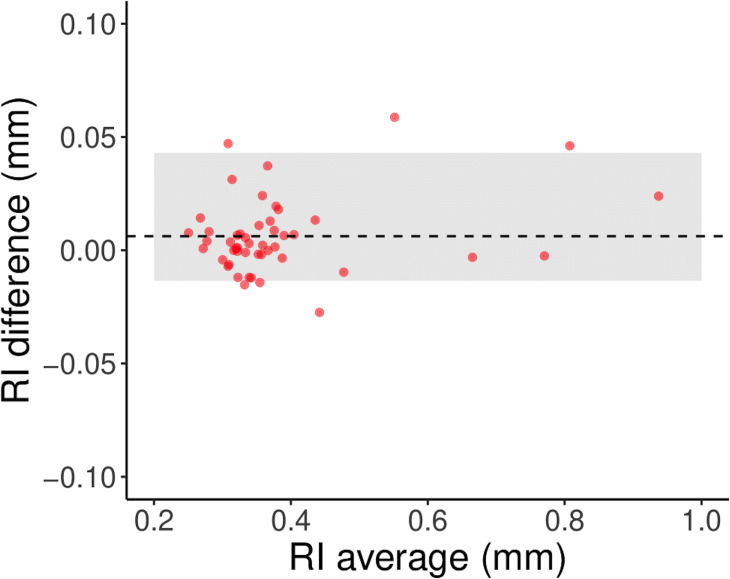
Bland-Altman plot showing the variability of the mean RI. The horizontal axis shows the average of the mean RI calculated from the measurements taken by each operator. The vertical axis reports its difference. The shaded area represents the 95% limits of agreement. The dashed line represents the mean difference. The figure reports the average and difference for 48 mean RI pairs, one pair for each scan.

There was an excellent inter-operator agreement for the mean RI between the two graders (Fig 3, 95% limits of agreement: -0.003, 0.043 mm). The average number of marked crossings per OCT-A scan was 65 ± 14 for Grader 1 and 82 ± 13 for Grader 2. The average inter-operator difference was 0.006.

**Fig 4:**
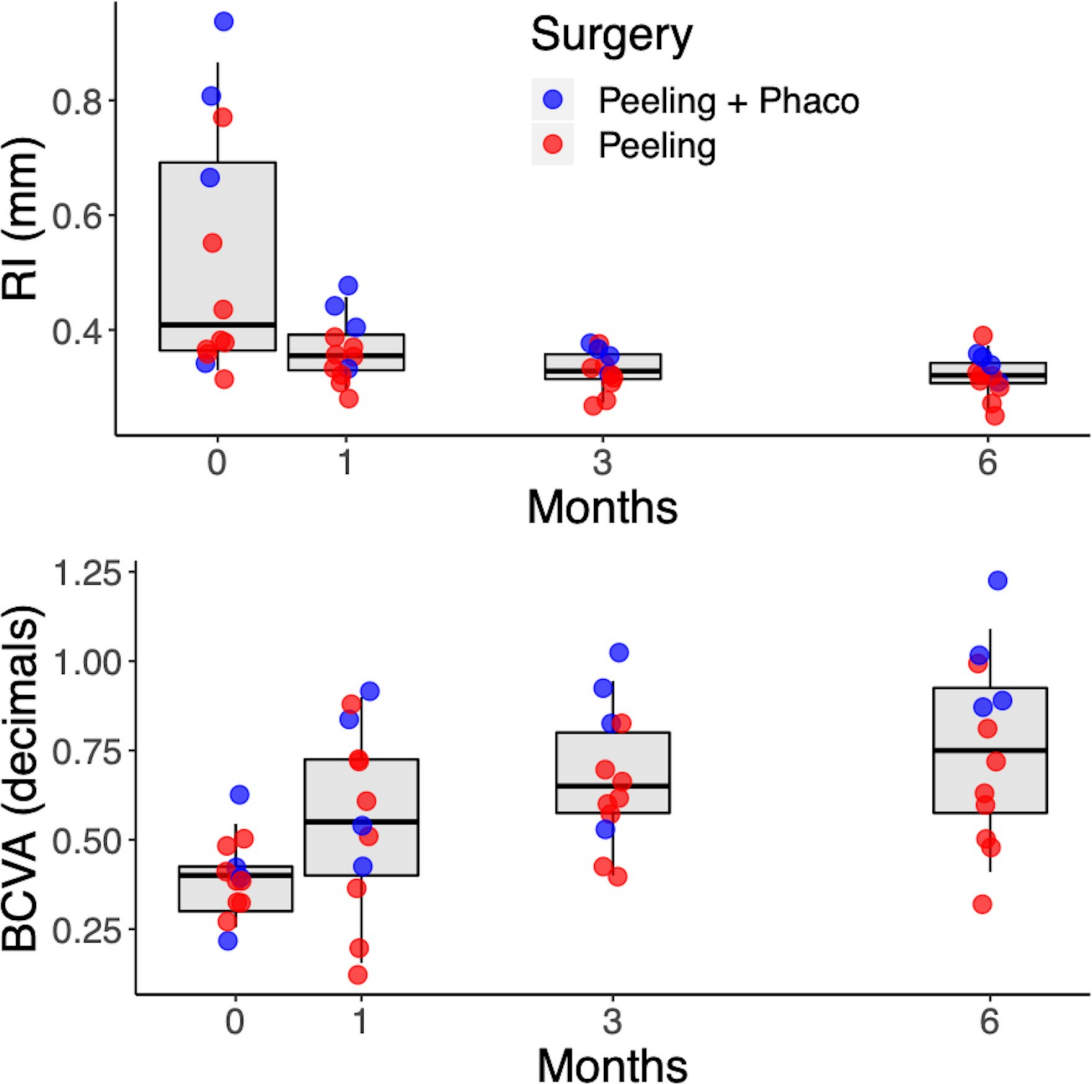
Changes in RI (top) and BCVA over time. The dots show the individual subjects, according to the surgical procedure they received.

BCVA increased over time (Fig 4) and was significantly better than baseline at all follow ups (Tables 1 and 2). An opposite trend was observed for the RI, which was maximum at baseline and then declined over time. The RI was also significantly smaller at all time points compared to baseline (Tables 1 and 2). There was no significant change in RI after the first month compared to 6 months (Table 2). However, the BCVA at the first month was still significantly smaller than at 6 months (p = 0.0190). There was a significant correlation between the RI and the BCVA (p < 0.001).

**Table 1:** BCVA and RI values at different time points (mean [95% confidence intervals]).

	BCVA (decimals)	RI (mm)
**Baseline**	0.39 [0.27–0.52]	0.53 [0.46–0.59]
**One month**	0.56 [0.43–0.69]	0.36 [0.30–0.43]
**Three months**	0.67 [0.54–0.79]	0.33 [0.26–0.40]
**Six months**	0.75 [0.62–0.88]	0.32 [0.25–0.39]

The p-values refer to comparisons with the baseline.

**Table 2:** P-values for the pairwise comparisons of BCVA and RI at different time points.

**BCVA (pairwise comparisons)**			
	**One month**	**Three months**	**Six months**
**Baseline**	0.0383	0.0006	< 0.0001
**One month**	-	0.1928	0.0190
**Three months**	-	-	0.1971
**RI (pairwise comparisons)**			
	**One month**	**Three months**	**Six months**
**Baseline**	0.0011	0.0001	0.0001
**One month**	-	0.8675	0.8675
**Three months**	-	-	0.8675

P-values were corrected for multiple testing using the Bonferroni-Holm method.

**Fig 5:**
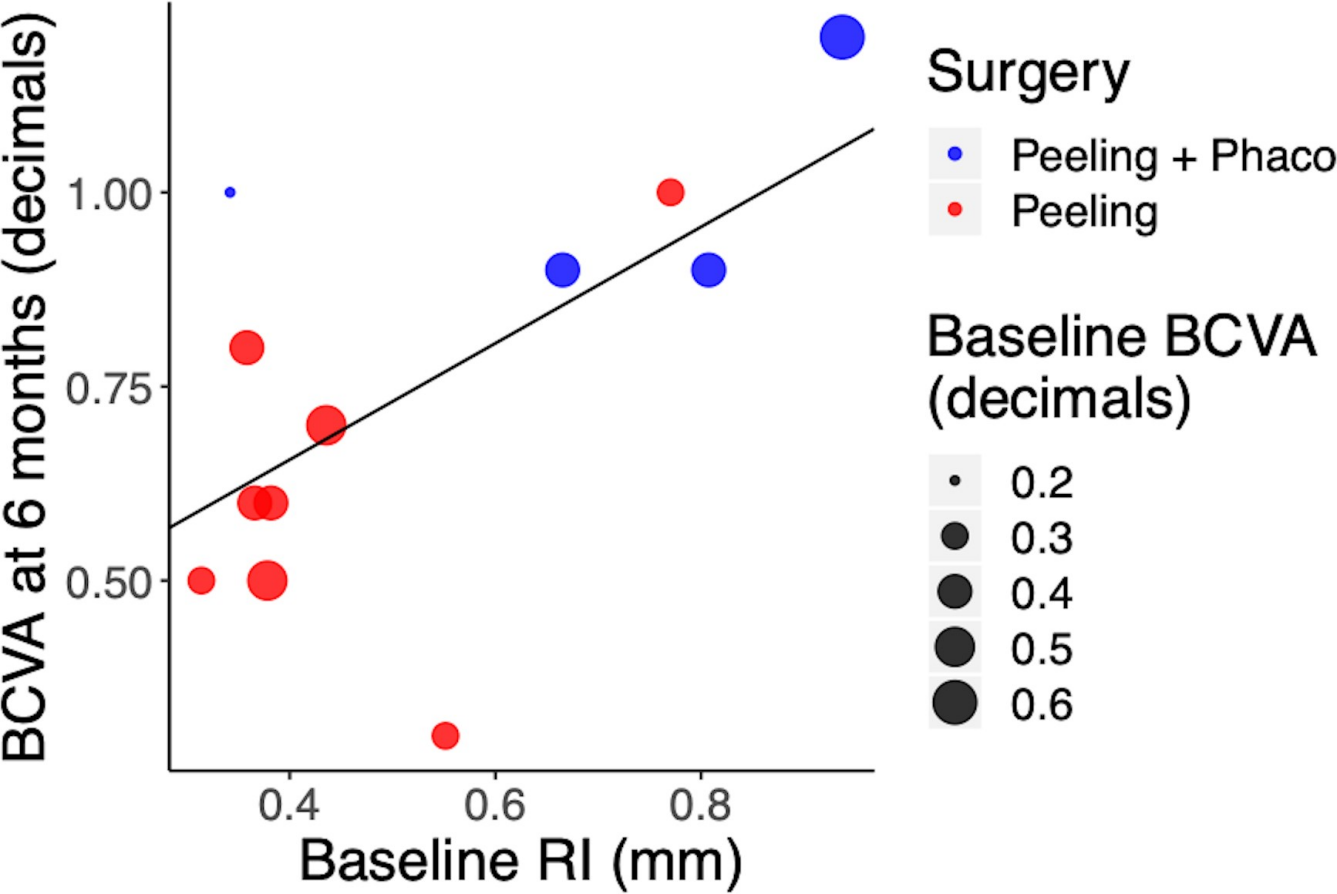
Relationship between the BCVA at 6 months and the RI at baseline. The size of the dots represents the BCVA at baseline.

There was no significant correlation between RI and BCVA at baseline (p = 0.268, correlation coefficient = 0.35). A larger baseline RI was shown to have significant correlation with a better postoperative BCVA at 6 months (Fig 5) when stratified by baseline BCVA (p = 0.0489, multivariate correlation coefficient = 0.62), meaning that the improvement in visual acuity was significantly correlated with retinal relaxation obtained after surgery.

## Discussion

ERM development involves a vast surface of the posterior pole [4]. However, all previous studies focused on bi-dimensional changes observed in the foveal region. In this study, we elaborated a method to evaluate in three-dimensions a vast area of the posterior pole.

Fibrocellular tissue contraction is a paramount feature of the fibrosis that occurs in ERM, which ultimately threatens sight [4]. This study introduces a simple and innovative method to measure retinal distortion caused by tangential traction. We measured the position shift of vascular structures after ERM peeling in progressive ERM, interpreted as a measure of released traction. RI was highest at baseline and reached final state at 1 month. No statistically significant difference (p <0.05) was noted with further assessments (Table 1). This finding may indicate that ERM peeling was successful in releasing traction allowing retinal layers distension and that this initial release accounted for the majority of the post-operative remodeling. Hartman et al. [19] reported, on average, a restoration of the normal anatomy 4 months after surgery. This is coherent with the idea that surgery offers an early release of traction and allows for retinal reorganization.

In this study, the scan at 6 months was considered as the maximum retinal relaxed state after surgery that we observed; however, our experimental evidence highlights that the RI does not change significantly after the first month and that the residual change between three and six months is minimal (0.01 average difference). Indeed, the major changes in both BCVA and RI happened at one month, indicating that retinal traction quantified by the RI is the major factor determining BCVA.

Furthermore, the present study found that BCVA was significantly better at every follow-up after surgery, with a gradual improvement in each visit; however, the change was small and not significant between 3 and 6 months. According to our results, the RI might not be sufficiently sensitive to predict subtle changes in BCVA after the first major retinal relaxation. Interestingly, we found a significant correlation between BCVA at 6 months and RI at baseline and in particular, cases with higher baseline RI (indicative here of larger relaxation after surgery) reached a higher BCVA at 6 months (Fig 5). Relaxation after surgery strictly depends on the grade of intra-retinal and epiretinal fibrosis, which is the result of the bond between Müller cells and ILM sustained by glial fibrillary acidic protein (GFAP) overexpression [20].

Traction has been recognized as an important prognostic factor in another study, that however focused on intra-retinal changes while measuring traction depth [21]. Moreover, many studies evaluated the relation between retinal layers morphology and BCVA, finding that the inner segment/outer segment (IS/OS) layer morphology can predict functional outcomes [22–24]. Nevertheless, intra-retinal modifications can be difficult to measure. On the other hand, qualitative descriptions of retinal changes can offer interesting insights but are ultimately limited by the narrow portion of retina considered and the subjectivity of clinical judgment.

On the contrary, our proposed index offers an objective measurement of traction release, providing a methodology to assess a successful surgery through the reduction in the RI. Even so, other features not described by the RI, such as changes to the IS/OS or to the photoreceptors, will likely need to be integrated in a multifactorial index to accurately describe the effects of ERMs on the retina and their functional consequences and this will be the objective of future work.

Of course, our current application is limited by the retrospective nature of the analysis. This implies that only RI reduction could be observed in our dataset by comparing the pre-operative state with the relaxed retina at 6 months after surgery, assumed to be at maximum relaxation. Nonetheless, this is not an intrinsic limitation of the methodology. Indeed, such a measure could be used in prospective observations to precisely track changes in retinal morphology over time. This latter aspect should be explored in a prospective study with long pre-operative follow-up series and will be the objective of future work.

To the best of our knowledge, this is the first study introducing a measurement for epiretinal traction and demonstrating its efficacy in the assessment of patients undergoing surgical treatment.

Our analysis is fraught by some limitations. The major hurdle for wide-scale clinical application is the need for manual landmark placement to detect vascular shifts therefore, automation of this process will be a crucial step in development of the technique. More sophisticated image analysis, including machine learning approaches, could be relatively fast and effective, but will likely need larger datasets to be implemented. The measurement of the local retinal thickness could also be automated with direct access to segmentation data, possibly aided by the use of freely available segmentation software [25, 26]. In addition, our analysis rests on the assumption that the built-in fundus tracking software used by the Spectralis SD-OCT was able to effectively register images in our follow-up scans. Despite being a proprietary technology whose details have not been disclosed, we can safely assume that the registration software relies heavily on vascular structures, which are the major features used for alignment of fundus images [27–29]. These structure are, however, continuously changing in the post-operative phase and this is indeed the basis for our analysis. Consequently, careful evaluation of alignment accuracy for this specific application is of paramount importance, with the possible need for the implementation of bespoke alignment algorithms. Nevertheless, in our dataset, we could not detect, at least by visual inspection, any major errors in the alignment process. Particularly, the optic nerve head and the major vessel branches were always aligned, except in the parafoveal region, where post-operative changes were indeed expected. One assumption of our index is that photoreceptors are anchored to the RPE and that no horizontal sliding of the retina occurs in the post-operative phase. We feel that this assumption is justified given the current understanding of RPE-photoreceptor adhesion [16], but specific evidence to support this in the post-operative phase is still lacking. Furthermore, we included patients undergoing combined surgery (Phaco-Peeling) could have influenced our measurement of BCVA results.

Finally, the small sample size could have limited our ability to detect subtle changes beyond the early post-operative phase. We highlight that at this stage RI can only be calculated based on the relaxed state at 6 months and therefore cannot be used to predict the outcome before surgery. Future research based on pre-operative longitudinal follow-ups will determine whether this index can accurately predict post-operative visual outcomes. Indeed, future work will focus on further development and validation of the technique on larger datasets, which will likely require a cooperative effort from multiple clinical centers.

## Conclusion

This proposed new method measures retinal stress induced by epiretinal traction, allowing a precise and comprehensive evaluation of the macular region and its changes. This study demonstrates that patients with higher release of traction after surgery reached a higher BCVA at 6 months. Therefore, tangential traction can be easily measured and correlated with functional outcomes. In the future, the integration of RI measurement into clinical imaging systems will enable a systematic three-dimensional assessment of epiretinal traction progression.

## Supporting information

S1 File (XLSX)
